# Surprising Gendered Age Differences in Rural Malawians’ Early COVID-19 Pandemic Prevention Efforts

**DOI:** 10.1093/geronb/gbae031

**Published:** 2024-03-08

**Authors:** Tyler W Myroniuk, Hans-Peter Kohler, Victor Mwapasa, James Mwera, Iliana V Kohler

**Affiliations:** Department of Public Health, University of Missouri, Columbia, Missouri, USA; Population Aging Research Center (PARC), University of Pennsylvania, Philadelphia, Pennsylvania, USA; Department of Community and Environmental Health, Kamuzu University of Health Sciences, Blantyre, Malawi; Invest in Knowledge, Zomba, Malawi; Population Studies Center, University of Pennsylvania, Philadelphia, Pennsylvania, USA; (Social Sciences Section)

**Keywords:** Africa, Family, Gender, Household composition, Infectious disease

## Abstract

**Objectives:**

We assess how age, the presence of mature adults aged 45+ years, and recent deaths in rural households are associated with coronavirus disease 2019 (COVID-19) preventative actions and the likelihood of getting vaccinated against the virus in Malawi during early stages of the pandemic.

**Methods:**

We draw upon data from 2,187 rural Malawians who participated in a 2020 COVID-19 Phone Survey. We estimate the log odds of engaging in “low-cost” and “high-cost” COVID-19 preventative actions based on age, gender, household composition, and recent household deaths. Low-cost prevention efforts were washing hands with soap and water frequently, avoiding close contact with people when going out, and avoiding shaking hands. High-cost actions included staying at home and decreasing time spent close to people not living in their household. We also estimate the chances of acquiring the COVID-19 vaccine in early stages of its availability.

**Results:**

Mature women (45+ years) in general and younger men (<45 years)—living with at least one mature adult in the household—were less likely than others to comply with low-cost actions. Mature men were more likely than younger men (<45 years) to take on high-cost actions. To some extent, individuals who experienced a recent family death were more likely to engage in high-cost COVID-19 preventative actions as well as getting vaccinated.

**Discussion:**

Gendered age differences in preventing the transmission of COVID-19 offer hints of larger social norms affecting protective efforts. The analyses also inform future COVID-19 public health outreach efforts in Malawi and other rural SSA contexts.

Malawi is a pertinent context to study coronavirus disease 2019 (COVID-19) prevention as it is a low-income country (LIC) where, unlike in high-income countries (HICs), the resources to combat the transmission of COVID-19 were initially, and continue to be, severely limited. Due to the airborne transmission of COVID-19 and limited availability of resources to address the pandemic, preventing its spread required Malawians to dramatically change their everyday interactions with individuals outside of their households to protect those within it (Banda et al., 2021). Known as the “Warm Heart of Africa,” Malawi has a strong cultural emphasis on being socially active (Myroniuk & Anglewicz, 2015); the country’s densely populated rural areas (National Statistical Office of Malawi, 2019) and community-based subsistence farming society contribute to this ethos. Malawians’ initial distrust and skepticism toward the government’s public health messaging hindered COVID-19 prevention efforts too (Chilanga et al., 2022; Kao et al., 2021).

Recent scholarship suggests that rural Malawians were able to more effectively combat COVID-19 in the early stages of the pandemic (Theu et al., 2022) than urban dwellers in part due to effective leadership and messaging from village leaders (Kohler et al., 2022). New evidence also suggests that Malawians’ COVID-19 prevention behaviors were linked to AIDS-related mortality that individuals witnessed a decade or more earlier (Anglewicz et al., 2023). It is *plausible* that rural Malawians—particularly “mature” adults 45+ years old—have become well-accustomed to managing the spread of infectious disease because they simply have survived the HIV/AIDS epidemic in addition to being accustomed to regularly managing outbreaks of other infectious diseases. Our novel research examines how COVID-19 was initially combatted by a mature adult population with high knowledge of the dangers of infectious diseases in a LIC setting.

## Conceptual Framework and Hypotheses

Rural Malawians tend to live in extended family households. Given the severe health risks that COVID-19 infection presented to older adults (age 45+ years; Anscombe et al., 2023)—which was found throughout the world too (Biswas et al., 2020; Starke et al., 2021)—the presence of mature adults in rural Malawian households may have altered pandemic behavior among its younger household members, as found in HICs (Stokes & Patterson, 2020; Voo et al., 2020). We assess how age, the presence of mature and older Malawians 45+ years in the household, and the experience of recent deaths in rural households are associated with COVID-19 preventative actions and the likelihood of getting vaccinated against the virus in earlier stages of the pandemic, respectively. Since distinct, gendered roles are a key feature of rural Malawian life and often disadvantage women—even in matrilineal kinship systems—regarding access to healthcare and in financial decision-making (Azad et al., 2020; Conroy, 2014; Gipson et al., 2010; Minton & Knottnerus, 2008), when possible, our analyses also stratified by gender.

Age stratification theory (Elder, 1994; Riley et al., 1972) guides our research, given that age is a “socially structured” place (Barken, 2019) and not only a biological one. As seen with HIV/AIDS research in sub-Saharan Africa (SSA), people of different generations have markedly different experiences combatting infectious diseases, the preventative strategies they take, and the social stigma around disease transmission (Golaz et al., 2017; Schatz et al., 2021; Zuch & Lurie, 2012).

First, we expect that the oldest Malawians (65+ years), and individuals in households with mature adults (45+ years), would be the most COVID-19-averse since they are the ones who have successfully navigated another infectious disease epidemic (HIV/AIDS) and been exposed to public health messaging the longest. Second, we expect that households that have experienced a recent death—for any reason—will engage in best COVID-19 preventative practices due to the universal importance of individual experiences, particularly with witnessing deaths, in shaping one’s health behaviors (Betancourt et al., 2016; Kapitány-Fövény, 2022).

## Method

### Data

Our analysis utilizes data from the Malawi Longitudinal Study of Families and Health (MLSFH), which is an ongoing population-based cohort study established in 1998 (Kohler et al., 2015, 2020). The study population lives overwhelmingly in rural communities in three districts in Malawi (Mchinji in the central, Rumphi in the northern, and Balaka in the southern regions). The original 1998 MLSFH study sample was drawn based on a probabilistic population sample. The study population has been augmented by enrolling adolescents, parents, new spouses, and most recently biological siblings of respondents in the later rounds of data collections. Comparisons of the MLSFH study population with the rural samples of the Malawi Demographic and Health surveys and the Integrated Household Survey (IHS3) show that the study population closely matches the rural subsample in the 2010 nationally representative IHS3 (Kohler et al., 2015, 2020).

To test our hypotheses, we combine the following MLSFH data: (1) the 2018 wave of the MLSFH Mature Adults Cohort (MLSFH-MAC) that includes respondents aged 45+ years; (2) the 2019 wave of MLSFH focused on study participants younger than age 45 years; (3) the MLSFH 2020 COVID-19 Phone Survey, which took place between early June and late August 2020 and captured the early phase of the pandemic; and (4) the 2021 MLSFH data collection, which took place between November and December 2021 and importantly included individuals’ COVID-19 vaccination statuses (the data identifies sibling pairs—where the COVID-19 vaccine information was collected—resulting in fewer longitudinally matched participants [*N* = 1,126]).

Thus, to evaluate COVID-19 preventative behaviors among Malawians, we draw upon data from a pool of 2,187 participants—living predominantly in rural areas—18 years and older who: (1) participated in the 2020 COVID-19 Phone Survey; and (2) at least one of the above mentioned MLSFH data rounds.

### Variables

Our three main outcome variables are: (1) Did the respondent engage in “low-cost” COVID-19 preventative actions? (1 = Yes, 0 = No); (2) Did the respondent engage in “high-cost” COVID-19 preventative actions? (1 = Yes, 0 = No); and (3) Did the respondent receive the COVID-19 vaccine? (1 = Yes, 0 = No)? We define “low-cost” and “high-cost” actions to prevent a COVID-19 infection following the approach by Kohler et al. (2022). More specifically, low-cost actions are defined as engaging in all the following general COVID-19 prevention efforts that would take little additional effort in daily life and would have minimal financial consequences associated with such efforts: washing hands, avoiding shaking hands, and avoiding close contacts with people outside of the household. High-cost measures include following all these prevention behaviors: staying at home and decreasing time spent with people outside of the household. These high-cost actions correspond to behaviors that bear high economic costs, are difficult to implement in subsistence agricultural societies, and, as a result, could more substantively affect one’s finances or social/economic lives.

For COVID-19 vaccination status, respondents were simply asked if they had been vaccinated for COVID-19—not the type of vaccine or dose. The rollout of COVID-19 vaccines in Malawi followed a similar approach as in other countries: in the initial phases of vaccination, the government prioritized healthcare workers, older adults, and other high-risk groups. By the end of 2021, the vaccine was available for everyone in the population, and the government implemented several programs for increased vaccination uptake including the “COVID-19 Vaccine Express” and “Vaccinate my Village” campaigns.

Our first primary predictor variable is if a respondent indicated that there was a household death in the last 2 years, depending on which MLSFH survey they participated in (2018 or 2019); this serves as a proxy for the immediacy of experiencing a recent death (position of the household family member did not matter in sensitivity analyses) but comes with the caveat that if one participated in 2018 than their household death in the previous 2 years refers to that time of survey versus in 2019, which is one year closer to the 2020 COVID survey data.

Our next primary predictor variables come from the 2020 COVID-19 Phone Survey: respondent age (<45 years, 45–64 years, 65+ years) to consider both age-based health risks in the COVID-19 pandemic and generationally different experiences with Malawi’s HIV/AIDS epidemic. For instance, HIV/AIDS infection prevalence is decreasing among younger Malawians while increasing among the older population, suggesting that more, older HIV-positive, individuals are surviving to older ages (Payne et al., 2023). At the same time, knowledge and prevention of HIV transmission are the highest they have been since the onset of the pandemic in the 1980s, due in part to a strong evidence-based response supported by Malawi’s government (Hargreaves et al., 2023).

Our final primary predictor variables are the number of household members 45+ years (0, 1, 2+) who are known as “mature adults,” and the number of older household members 65+ years (0, 1, 2+), with both excluding respondents in their respective age categories.

Our control variables are gender, main source of COVID-19 information (local health personnel versus other sources), estimated COVID-19 prevalence in the area (in deciles), marital status (married versus not), educational attainment (completed secondary versus not), and region within Malawi (central, south, or north). In our final set of estimates predicting COVID-19 vaccination, engaging in all low-cost and all high-cost COVID-19 prevention actions acts as controls (in separate blocks of models). Day and month fixed effects were ultimately excluded as controls since they did not substantively change any estimates while adding unnecessary model parameters.

### Modeling

All our logistic regression models—using the variables described above—are effectively cross-sectional, even though we use information from waves collected on data from prior years to predict outcomes in the present through our combined data set; we do not include repeated measures in our analyses. Our first two sets of modeling are identical, except for the outcome variables. We estimate the log odds of respondents engaging in all low-cost COVID-19 preventative actions and high-cost COVID-19 preventative actions in gender-stratified, then gender- *and* age-stratified (<45, 45–64 years old) models. Stratifying by gender is ideal and conventional, if possible, because of the distinctly gendered roles in everyday rural Malawian life, whether in patriarchal or matriarchal inheritance systems. Substratifying by age is necessary to effectively remove bias when estimating how household composition *based on age* is associated with COVID-19 preventative actions. For example, individuals ages 45–64 are not included in estimates of how the number of 45+ year-olds in a household is associated with their own COVID-19 preventative behavior as those 45–64 would effectively be double-counted in the estimating procedure. There are too few respondents aged 65 years and older to run reliable, age-stratified regressions for this group. Our third set of models, predicting the log odds of having received a COVID-19 vaccine, is not stratified by gender, but stratified by age (<65 only) due to insufficient cell sizes.

In our regression analyses, we report the 95% confidence intervals and discrete *p* value cutoffs. We also include an indicator of a lower threshold (*p* < .10, denoted by #) for conventional statistical significance cutoffs as well, for additional context, which should be interpreted cautiously with respect to the sample size and number of parameters estimated in each regression model.

## Results

Table 1 shows that most respondents engaged in all low-cost COVID-19 preventative actions in the early days of the pandemic (80.3%). However, fewer engaged in all high-cost preventative actions (55.9%). By November/December 2021—over a year later—only 26.2% of respondents reported receiving a COVID-19 vaccine but this was in part due to the timing of vaccine rollout in Malawi and limited supply in the beginning of the rollout. Based on the 2018/2019 surveys, at least 1 in 10 of the respondents indicated that a household member had died in the previous 2 years (11.8%). In 2020, most respondents lived in a household with at least one member who was aged 65+ years (59.4%) and most lived in a household with at least one member who was aged 45+ years (94.1%). The largest group of respondents were under 45 years (45.1%), followed by those 45+ and less than 65 (41.8%), and finally those aged 65+ (13.1%).

**Table 1.** Descriptive Statistics

Variable	*n*	%	$\bar{x}$ (*SD*)
*Outcome variables*			
Engaged in all low-cost COVID-19	2,182		
Preventative actions			
Yes		80.3	
No		19.7	
Engaged in all high-cost COVID-19	2,182		
Preventative actions			
Yes		55.9	
No		44.1	
Received COVID-19 vaccine	1,126		
Yes		26.2	
No		73.8	
*Independent variables*			
Household death in the last 2 years	2,180		
Yes		11.8	
No		88.2	
# Household members 65+	2,187		
0		40.6	
1		39.0	
2+		20.4	
# Household members 45+	2,187		
0		5.9	
1		21.2	
2+		72.9	
Age	2,187		
<45		45.1	
45–64		41.8	
65+		13.1	
*Control variables*			
Gender	2,187		
Male		41.0	
Female		59.0	
Source of COVID-19 info	2,181		
Local health personnel		56.8	
Other sources		43.2	
Estimated COVID-19 prevalence in area (deciles)	2,098		7.8 (13.8)
Marital status	2,187		
Married		84.7	
Not married		15.3	
Education	2,187		
Completed secondary		19.8	
Did not complete secondary		80.2	
Region	2,187		
Central		35.8	
South		25.7	
North		38.5	

*Notes:* COVID-19 = coronavirus disease 2019; *SD* = standard deviation.

The log odds of engaging in all low-cost COVID-19 preventative actions are estimated in Table 2’s gender- and age-stratified models. Models 1 and 2 show that women 45–64 years old (*p* < .05) and 65+ years old (*p* < .05) were both *less likely* than those under 45 years to engage in all low-cost actions, but these two age groups were not statistically different from one another. There was no difference in the log odds of engaging in all low-cost actions, whether female or male respondents reported a recent household death or not (across all models, including those stratified by age). We also find that for men under 45 years old, those who had at least one member 45 years or older in the household were less likely to engage in low-cost preventative actions (*p* < .01; *p* < .05, model 8) than those who had no one 45 years and older in the household; this contrasts with our expectations.

**Table 2.** Logistic Regressions Predicting Engaging in All Low-Cost COVID-19 Preventative Actions (Gender Stratified)

Variable	Model 1	Model 2	Model 3	Model 4	Model 5	Model 6	Model 7	Model 8
	Female	Male	Female	Male	Female	Male	Female	Male
	All ages	All ages	Age < 45	Age < 45	Age 45–64	Age 45–64	Age < 45	Age < 45
Household death last 2 years (ref. not)	0.17 [−0.28, 0.62]	0.27 [−0.32, 0.86]	0.24 [−0.60, 1.08]	0.03 [−1.11, 1.16]	0.51 [−0.15, 1.16]	0.22 [−0.64, 1.07]	0.23 [−0.62, 1.08]	0.27 [−0.90, 1.43]
# Household members 65+ (ref. 0)								
1			0.19 [−0.29, 0.67]	−0.08 [−0.70, 0.53]	−0.15 [−0.65, 0.35]	−0.16 [−0.72, 0.41]		
2+			0.06 [−0.55, 0.66]	0.03 [−0.81, 0.86]	0.00 [−0.69, 0.69]	−0.60 [−1.39, 0.19]		
# Household members 45+ (ref. 0)								
1							0.01 [−0.77, 0.79]	−1.95^**^ [−3.18, −0.71]
2+							−0.04 [−0.78, 0.70]	−1.31^*^ [−2.56, −0.07]
Age (ref. <45)								
45–64	−0.36^*^ [−0.67, −0.04]	−0.09 [−0.49, 0.31]						
65+	−0.60^*^ [−1.10, −0.11]	−0.04 [−0.55, 0.48]						
Source of COVID-19 info—local health personnel (ref. other)	0.13 [−0.16, 0.42]	0.61^***^ [0.27, 0.96]	0.09 [−0.35, 0.52]	0.14 [−0.43, 0.71]	0.22 [−0.24, 0.68]	0.99^***^ [0.47, 1.51]	0.09 [−0.34, 0.53]	0.10 [−0.48, 0.68]
Estimated COVID-19 prevalence in area (deciles)	0.00	0.00	0.00	0.01	0.00	−0.00	0.00	0.01
Married (ref. not)	−0.07 [−0.42, 0.29]	−0.06 [−0.83, 0.71]	−0.20 [−0.82, 0.41]	0.23 [−0.69, 1.15]	0.02 [−0.52, 0.57]	0.14 [−1.55, 1.82]	−0.20 [−0.81, 0.42]	0.28 [−0.65, 1.21]
Completed secondary education (ref. not)	0.24 [−0.23, 0.71]	0.32 [−0.11, 0.76]	0.20 [−0.36, 0.75]	−0.08 [−0.68, 0.51]	0.19 [−0.75, 1.14]	0.78^#^ [−0.06, 1.61]	0.20 [−0.36, 0.75]	−0.14 [−0.75, 0.48]
Region (ref. Central)								
South	0.69^***^ [0.31, 1.07]	0.16 [−0.30, 0.63]	0.40 [−0.15, 0.96]	0.67 [−0.20, 1.55]	0.99^**^ [0.39, 1.58]	−0.00 [−0.69, 0.69]	0.40 [−0.16, 0.95]	0.67 [−0.21, 1.54]
North	0.56^***^ [0.23, 0.90]	−0.31 [−0.71, 0.09]	0.41 [−0.09, 0.91]	−0.06 [−0.68, 0.57]	0.73^**^ [0.21, 1.25]	−0.50 [−1.12, 0.11]	0.42 [−0.08, 0.92]	−0.04 [−0.69, 0.61]
Constant	1.15^***^ [0.69, 1.61]	1.17^**^ [0.35, 1.99]	1.35^***^ [0.61, 2.09]	1.12^*^ [0.07, 2.16]	0.57^#^ [−0.09, 1.22]	0.94 [−0.78, 2.66]	1.43^**^ [0.50, 2.37]	2.50^**^ [0.98, 4.02]
Observations	1,224	872	624	339	487	386	624	339
Pseudo *R*^2^	0.024	0.022	0.011	0.018	0.033	0.061	0.010	0.063

*Notes*: COVID-19 = coronavirus disease 2019; ref. = reference. Log odds presented. 95% Confidence intervals in brackets.

^#^ *p* < .10.

^*^ *p* < .05.

^**^ *p* < .01.

^***^ *p* < .001.

In Table 3, women—generally—*who reported a recent household death* had higher log odds of engaging in all high-cost COVID-19 preventative actions compared to those who did not report a recent household death (*p* < .10, model 1). More specifically, this is also evidence for this relationship for women under 45 years old (*p* < .10, model 7 but not model 3) and men under 45 years old (*p* < .10, *p* < .05, models 4 and 8, respectively). Men ages 45–64 and 65 years and above had higher log odds of engaging in all high-cost preventative actions than those under 45 years (*p* < .05; *p* < .001, model 2). Yet again, though, there is evidence that the presence of older individuals in a household was associated with lower chances of engaging in all high-cost preventative actions. Women ages 45–64 *in a household with* two or more members 65+ years had lower log odds of engaging in all high-cost preventative actions than those with only one or none in the house (*p* < .10, model 5). Further, men under 45 years who had only one member 45 years and older were less likely to engage in all high-cost preventative actions than those who did not have anyone 45 years and older in their household (*p* < .05, model 8).

**Table 3.** Logistic Regressions Predicting Engaging in All High-Cost COVID-19 Preventative Actions (Gender Stratified)

Variable	Model 1	Model 2	Model 3	Model 4	Model 5	Model 6	Model 7	Model 8
	Female	Male	Female	Male	Female	Male	Female	Male
	All ages	All ages	Age < 45	Age < 45	Age 45–64	Age 45–64	Age < 45	Age < 45
Household death last 2 years (ref. not)	0.34^#^ [−0.03, 0.71]	0.23 [−0.22, 0.68]	0.53 [−0.12, 1.19]	0.83^#^ [−0.05, 1.70]	0.06 [−0.44, 0.56]	−0.07 [−0.75, 0.60]	0.57^#^ [−0.09, 1.23]	1.03^*^ [0.12, 1.94]
# Household members 65+ (ref. 0)								
1			0.24 [−0.13, 0.61]	−0.12 [−0.62, 0.37]	−0.21 [−0.63, 0.21]	0.26 [−0.20, 0.71]		
2+			0.20 [−0.28, 0.67]	0.42 [−0.22, 1.07]	−0.50^#^ [−1.07, 0.06]	−0.37 [−1.09, 0.34]		
# Household members 45+ (ref. 0)								
1							0.24 [−0.35, 0.82]	−0.70^*^ [−1.36, −0.04]
2+							0.40 [−0.16, 0.95]	0.02 [−0.60, 0.64]
Age (ref. <45)								
45–64	−0.05 [−0.30, 0.21]	0.40^*^ [0.08, 0.72]						
65+	0.31 [−0.14, 0.76]	0.73^***^ [0.32, 1.15]						
Source of COVID-19 info—local health personnel (ref. other)	−0.03 [−0.27, 0.21]	0.36^**^ [0.09, 0.64]	−0.17 [−0.51, 0.17]	0.10 [−0.36, 0.55]	0.14 [−0.24, 0.53]	0.68^**^ [0.26, 1.10]	−0.17 [−0.51, 0.17]	0.10 [−0.35, 0.56]
Estimated COVID-19 prevalence in area (deciles)	0.01^**^ [0.00, 0.02]	0.01^*^ [0.00, 0.02]	0.02^**^ [0.00, 0.03]	0.00 [−0.02, 0.02]	0.01 [−0.01, 0.02]	0.03^**^ [0.01, 0.05]	0.02^*^ [0.00, 0.03]	0.00 [−0.02, 0.02]
Married (ref. not)	−0.03 [−0.33, 0.26]	−0.24 [−0.84, 0.37]	0.20 [−0.25, 0.65]	0.32 [−0.49, 1.13]	−0.04 [−0.50, 0.41]	−0.51 [−1.90, 0.88]	0.17 [−0.28, 0.62]	0.32 [−0.49, 1.14]
Completed secondary education (ref. nt)	0.16 [−0.19, 0.51]	0.19 [−0.15, 0.53]	0.33 [−0.09, 0.74]	−0.25 [−0.73, 0.23]	−0.24 [−0.95, 0.48]	0.48 [−0.12, 1.09]	0.34 [−0.08, 0.76]	−0.29 [−0.78, 0.19]
Region (ref. Central)								
South	0.45^**^ [0.14, 0.76]	0.03 [−0.32, 0.39]	0.58^*^ [0.13, 1.02]	−0.51 [−1.14, 0.12]	0.44^#^ [−0.05, 0.94]	0.08 [−0.46, 0.62]	0.58^*^ [0.14, 1.02]	−0.47 [−1.10, 0.15]
North	0.24^#^ [−0.04, 0.51]	−0.03 [−0.35, 0.30]	0.17 [−0.21, 0.55]	0.35 [−0.17, 0.87]	0.36 [−0.10, 0.81]	−0.19 [−0.69, 0.31]	0.17 [−0.21, 0.56]	0.31 [−0.22, 0.84]
Constant	0.17 [−0.21, 0.55]	−0.57^#^ [−1.22, 0.09]	−0.14 [−0.70, 0.42]	−0.77^#^ [−1.67, 0.13]	0.28 [−0.30, 0.85]	−0.19 [−1.61, 1.23]	−0.31 [−1.02, 0.39]	-0.51 [−1.51, 0.48]
Observations	1,225	873	624	339	487	386	624	339
Pseudo *R*^2^	0.015	0.023	0.027	0.026	0.013	0.052	0.028	0.039

*Notes*: COVID-19 = coronavirus disease 2019; ref. = reference. Log odds presented. 95% Confidence intervals in brackets.

^#^ *p* < .10.

^*^ *p* < .05.

^**^ *p* < .01.

^***^ *p* < .001.

The log odds of receiving a COVID-19 vaccine are estimated in Table 4. Most notably, reporting a recent household death (*p* < .05, all models) was positively associated with COVID-19 vaccination. Though seen only in all-age models (models 1 and 4), engaging in all low-cost (*p* < .05, model 1) and high-cost COVID-19 preventative actions (*p* < .10, model 4) were also predictive of COVID-19 vaccination, as seen in model 1 (gender-pooled, all ages). When considering only those less than 65 years old (models 5 and 6), a recent household death were more strongly associated with COVID-19 vaccination (*p* < .05) even though the effects of engaging in all high-cost preventative actions was no longer substantial. Age *itself* was not robustly linked to the decision to get vaccinated as those 65+ years in model 1 had higher log odds of getting vaccinated than younger individuals, but this effect was not found in model 4—where the control for engaging in all high-cost preventative actions was included instead of all low-cost preventative actions. The presence of older household members (in model 3) was not associated with COVID-19 vaccination for those under 65 years old. Thus, the recency of death within a household was the most robust factor associated with the log odds of COVID-19 vaccination.

**Table 4.** Logistic Regressions Predicting COVID-19 Vaccination (Gender-Pooled)

Variable	Model 1	Model 2	Model 3	Model 4	Model 5	Model 6
	Gender pooled	Gender pooled	Gender pooled	Gender pooled	Gender pooled	Gender pooled
	All ages	Age < 65	Age < 65	All ages	Age < 65	Age < 65
Household death last 2 years (ref. not)	0.43^*^ [0.05, 0.82]	0.53^*^ [0.10, 0.96]	0.54^*^ [0.11, 0.97]	0.42^*^ [0.04, 0.80]	0.53^*^ [0.10, 0.96]	0.54^*^ [0.11, 0.98]
# Household members 65+ (ref. 0)						
1			0.01 [−0.35, 0.37]			−0.01 [−0.37, 0.35]
2+			0.08 [−0.42, 0.58]			0.11 [−0.39, 0.61]
Engaged in all low-cost COVID-19 preventative actions	0.45^*^ [0.08, 0.82]	0.33 [−0.09, 0.75]	0.33 [−0.09, 0.75]			
Engaged in all high-cost COVID-19 preventative actions				0.28^#^ [−0.02, 0.58]	0.27 [−0.07, 0.60]	0.28 [−0.06, 0.61]
Age (ref. <45)						
45–64	0.37 [−0.21, 0.95]	0.35 [−0.22, 0.92]	0.35 [−0.22, 0.93]	0.37 [−0.21, 0.94]	0.34 [−0.23, 0.92]	0.35 [−0.23, 0.92]
65+	0.56^#^ [−0.08, 1.19]			0.52 [−0.11, 1.16]		
Male (ref. female)	0.24 [−0.07, 0.56]	0.21 [−0.14, 0.55]	0.22 [−0.14, 0.57]	0.29^#^ [−0.03, 0.60]	0.25 [−0.10, 0.60]	0.26 [−0.09, 0.61]
Source of COVID-19 info—local health personnel (ref. other)	0.18 [−0.12, 0.48]	0.26 [−0.08, 0.61]	0.26 [−0.08, 0.61]	0.20 [−0.10, 0.50]	0.28 [−0.07, 0.62]	0.27 [−0.07, 0.62]
Estimated COVID-19 prevalence in area (deciles)	−0.00 [−0.01, 0.01]	−0.01 [−0.02, 0.01]	−0.01 [−0.02, 0.00]	−0.00 [−0.01, 0.01]	−0.01 [−0.02, 0.00]	−0.01 [−0.02, 0.00]
Married (ref. not)	−0.07 [−0.51, 0.36]	0.04 [−0.49, 0.56]	0.03 [−0.49, 0.56]	−0.07 [−0.51, 0.36]	0.03 [−0.49, 0.56]	0.03 [−0.50, 0.56]
Completed secondary education (ref. not)	0.56^*^ [0.13, 0.98]	0.42^#^ [−0.06, 0.89]	0.42^#^ [−0.06, 0.89]	0.58^**^ [0.16, 1.00]	0.43^#^ [−0.05, 0.90]	0.43^#^ [−0.05, 0.91]
Region (ref. Central)						
South	−1.18^***^ [−1.65, −0.71]	−0.98^***^ [−1.50, −0.47]	−0.98^***^ [−1.50, −0.47]	−1.17^***^ [−1.64, −0.70]	−0.97^***^ [−1.48, −0.45]	−0.97^***^ [−1.49, −0.46]
North	0.44^**^ [0.12, 0.77]	0.39^*^ [0.02, 0.76]	0.39^*^ [0.02, 0.75]	0.45^**^ [0.13, 0.77]	0.40^*^ [0.03, 0.76]	0.39^*^ [0.03, 0.76]
Constant	−2.04^***^ [−2.81, −1.27]	−2.01^***^ [−2.85, −1.17]	−2.03^***^ [−2.89, −1.17]	−1.87^***^ [−2.61, −1.13]	−1.92^***^ [−2.72, −1.12]	−1.94^***^ [−2.76, −1.11]
Observations	1,060	837	837	1,062	837	837
Pseudo *R*^2^	0.082	0.067	0.067	0.080	0.067	0.067

*Notes*: COVID-19 = coronavirus disease 2019; ref. = reference. Log odds presented. 95% Confidence intervals in brackets.

^#^ *p* < .10.

^*^ *p* < .05.

^**^ *p* < .01.

^***^ *p* < .001.

## Discussion

The gendered age differences between preventing the transmission of COVID-19 through low-cost actions and high-cost actions offer hints of larger, social norms affecting protective efforts even if we cannot pinpoint the mechanisms of these phenomena in this analysis.

First, our results indicate that younger men (<45 years) *with at least one mature adult in the household (compared to none)* and mature women (45+ years) *in general* were less likely to comply with all low-cost COVID-19 preventative actions—the most basic precautions. Men under 45 years *may have* taken on the risks of daily activities—such as work where social distancing was not possible or work sites that were not conducive to maintain hand hygiene—outside of their homes and rural properties so that those 45+ years did not have to, given their increased risk of adverse COVID-19 health outcomes; these would be analogous to family efforts to prevent COVID-19 transmission throughout the world. Similar effects were seen regarding engaging in all high-cost COVID-19 preventative actions for women 45–64 who had 65+ year-olds within their households, as well as men under 45 years who had one 45+ year-old household member; age, gender, and the ages of household members are associated with COVID-19 prevention efforts but not uniformly.

Second, for rural Malawian women 45–64 years and 65+ years old, day-to-day domestic duties and expected caregiving of younger and older family members (Minton & Knottnerus, 2008) could have stifled efforts to engage in low-cost prevention practices. The results estimating engaging in all high-cost COVID-19 preventative actions add support to this gendered possibility by the other side of filial expectations: mature men were more likely than younger men (<45 years) to (a) stay at home and (b) decreased time spent close to persons not living in their household because of social norms providing deference (and less responsibility for domestic duties) to mature men (Swidler, 2013). Third, the limited availability of the COVID-19 vaccine until late 2021 likely prevented any association between participant age and the age of household members from occurring; those who could get vaccinated did because of its paramount public health importance. In short, our first hypothesis—which predicted that those 65+ would be most COVID-19 averse through their actions, has less evidence to support it than expected, in part due to gendered differences.

We expected that individuals who experienced a recent family death would be more likely to engage in low-cost and high-cost COVID-19 preventative actions, as well as getting vaccinated. Overall, there was some evidence of this relationship. Women, overall, and men under 45 years who experienced a recent household death were more likely to engage in all high-cost preventative actions, though this may be the case for women under 45 years too (Table 3, model 7). *Anyone* who experienced a recent household death was more likely to get vaccinated for COVID-19 than those who did not. Thus, there was some support for our second hypothesis. It is reasonable that the combination of relatively low life expectancy (even with Malawi’s population starting to age), high mortality due to infectious diseases prior to COVID-19 (Allain et al., 2017), and COVID-19 itself put death at the forefront of most Malawians’ thoughts during the early days of the pandemic; sorting out these processes is important for future research. Moreover, more qualitative and quantitative research is necessary to uncover gender and age differences in response to health shocks, including how official messaging can be adjusted to reach all genders and age groups.

Unfortunately, our data do not allow for causal inferences, even if temporal elements—such as the timing of household deaths—exist. The measured recent household deaths are not equally spaced for all participants too, due to the nature of prior MLSFH data collection; such deaths are self-reported, and even with interviewer prompting, participants’ self-reports of what constitutes a household can be flexible in African settings where extended family living arrangements are common (Guyer, 1981; Hosegood et al., 2005).

## Conclusion

Unlike in high-income countries where extensive efforts were taken to protect the oldest individuals from COVID-19 transmission—because of the health infrastructure and care available—the oldest Malawians (65+) did not have the same advantages protecting them from COVID-19 compared to younger Malawians; social norms, socioeconomic challenges, *and* a lack of formal health infrastructure undoubtedly played a role in this. More broadly, the gender discrepancies in COVID-19 prevention outcomes highlight the complicated role that gender plays in health management. Future inquiries should not be limited only to LICs like Malawi, but whether there is equality in infectious disease prevention behavior in HICs as well. Lastly, this article can inform future COVID-19 public health outreach efforts in Malawi and other rural SSA contexts, given the need for continued vaccination and prevention as COVID-19 immunity wanes and rural areas experience population aging.
